# Changes in smoking patterns after HIV diagnosis or antiretroviral treatment initiation: a global systematic review and meta-analysis

**DOI:** 10.1186/s40249-020-00644-z

**Published:** 2020-04-16

**Authors:** Jobert Richie Nansseu, Dalhia Noelle Tounouga, Jean Jacques Noubiap, Jean Joel Bigna

**Affiliations:** 1 Department of Public Health, Faculty of Medicine and Biomedical Sciences of the University of Yaoundé I, Yaoundé, Cameroon; 2 Department for the Control of Disease, Epidemics and Pandemics, Ministry of Public Health, Yaoundé, Cameroon; 3 Centre for Heart Rhythm Disorders, University of Adelaide and Royal Adelaide Hospital, Adelaide, Australia; 4 Department of Epidemiology and Public Health, Centre Pasteur of Cameroon, PO Box 1274, Yaoundé, Cameroon; 5 School of Public Health, Faculty of Medicine, University of Paris Sud XI, Le Kremlin Bicêtre, France

**Keywords:** Smoking, HIV, AIDS, Tobacco, Global health

## Abstract

**Background:**

Tobacco consumption is more life-threatening in people living with human immunodeficiency virus (HIV) than within the general population; therefore, people living with HIV (PLWH) should be highly motivated to take action towards quitting smoking at or after HIV diagnosis. The aim of this systematic review with meta-analysis was to investigate changes in smoking habits among PLWH over time.

**Main text:**

We considered prospective and retrospective cohort studies including PLWH aged 15 years and above, which have measured the prevalence of tobacco smoking (current, former or never) at study initiation and completion, and published between January 1, 2000 and April 15, 2018 without language or geographical restriction. We searched PubMed, EMBASE, Web of Science, Africa Journal Online, and Global Index Medicus. We used a random-effects model to pool data. Nine studies were included. The proportion of current and former smokers decreased slightly over time, around 2.5 and 3.8%, respectively. However, the proportion of never smokers decreased sharply by 22.5%, and there were 2.1 and 1.5% PLWH who shifted from never and former smoking to current smoking, respectively. On the other hand, 10.5% PLWH shifted from current to former smoking, 7.1% tried to quit tobacco consumption but failed, and 10.1% stayed in the “never smoking” category over time.

**Conclusions:**

PLWH seem not to change positively their smoking habits towards quitting tobacco consumption. There is urgent need to increase actions aimed at helping this vulnerable population to quit tobacco consumption, including individually tailored therapeutic education, psychosocial and pharmacologic supports.

## Background

Tobacco constitutes one of the leading causes of preventable deaths worldwide, killing over 7 million people each year among whom more than 80% occur in developing countries [1]. In addition, tobacco consumption accounts for almost 182 million disability-adjusted life year [1, 2]. Tobacco consumption increases the risk of developing chronic diseases such as cancers, cardiovascular diseases, chronic infections and chronic pulmonary diseases, particularly in people living with human immunodeficiency virus (HIV) [3–5]. In these populations specifically, more than 70% of whom are living in Africa [6], smoking is associated with a more rapid progression of the HIV disease with an increased likelihood of HIV-related complications, decreased adherence to antiretroviral therapy (ART), and lowered virological and immunological responses to ART [3, 4, 7–11].

Studies have reported that smoking tobacco is a highly prevalent behaviour among people living with HIV (PLWH), with an average of 16–28 cigarettes smoked per day among those who smoke, which is an indicator of high nicotine dependence [10, 12–16]. Furthermore, the prevalence of smoking among PLWH is at least 1.3 folds higher (range 40–74%) than in the general population (range 19–31%) [10, 11, 13, 17, 18]. Therefore, smoking cessation should be considered as a priority by HIV care providers.

Strikingly, reasons for this higher prevalence of smoking among PLWH have not been well elucidated. Some evidence indicates that tobacco use among PLWH may be associated with some factors including substance use disorders (heroin, cocaine, marijuana, crack, heavy alcohol drinking), socioeconomic factors (unemployment, lower educational level, lower income), mental disorders or lack of access to health services, housing, and transportation [7, 10, 12, 15, 19, 20]. As far as the Health Promotion Model in HIV care is concerned, knowing one’s HIV status is projected to increase self-motivation towards the control of one’s health and then, motivate self-appraisal of health risks [21]. Accordingly, when a PLWH starts accepting his/her condition as serious and life-threatening, he/she may be motivated to take action in order to control the disease process by quitting unhealthy habits including smoking. Therefore, it is expected that positive smoking behaviour changes would occur in the course of HIV-infection. We designed and conducted a systematic review and meta-analysis to summarize evidence on the changes in smoking patterns after HIV diagnosis or ART initiation.

## Main text

### Methods

#### Design

This systematic review with meta-analysis was reported following the Meta-analysis Of Observational Studies in Epidemiology (MOOSE) guidelines (Table S1). This review was registered in the PROSPERO International Prospective Register of systematic reviews, registration number CRD42019123969. The Centre for Reviews and Dissemination guidelines were used as a reference for the methodology of this review [22].

#### Criteria for considering studies for the review

We considered prospective and retrospective cohort studies, before-and-after studies and control arm of randomized controlled trials which have assessed changes in smoking behaviours among PLWH. Studies had to include global adult (> 15 years) populations living with HIV infection who have experienced any change in their smoking habits at the time of HIV diagnosis or ART initiation. These changes might have been from current smoker (people who actually smoke) to former smoker (people who smoked and who stopped), from former smoker to current smoker, from former smoker to current smoker, or from never-smoker (people who have never smoked) to current smoker. Studies included in this review had to measure the prevalence (or enough data to compute this estimate) of tobacco smoking pattern (current, former, and never) at least two times (at initiation and completion). Studies lacking primary data or explicit method description were excluded, if after contacting authors at least twice the information was not provided. We also excluded studies wherein HIV-negative individuals had been included without the possibility to extract data only for HIV-infected people. Similarly, we did not consider cross-sectional or case–control studies, letters, reviews, commentaries, editorials, case reports, or case series.

#### Search strategy used to identify relevant studies

A comprehensive search of databases was performed by a review author to identify all relevant articles published from January 1, 2000 to April 19, 2018 regardless of either the language of publication or the geographical location. The following databases were screened: MEDLINE through PubMed, Excerpta Medica Database, Web of Science, Africa Journal Online, and Global Index Medicus. A predefined strategy using combination of relevant terms and their variants was used. Both text words and medical subject heading terms were used, including “HIV”, “AIDS”, “smoking”, “tobacco” and “cigarette”. The literature search strategy was adapted to suit each database. The search strategy conducted in PubMed is shown in Table S2. To identify other sources, reference lists of eligible articles and relevant reviews were manually scanned.

#### Selection of included studies

We developed and piloted a screening guide to make sure that all inclusion criteria were adhered to and consistently applied by all review authors. Two authors independently reviewed all identified citations on the basis of their titles and abstracts; then, the same authors independently assessed the full-texts of records deemed relevant or potentially relevant for eligibility. Any discrepancy between these authors was resolved through discussion and consensus, or arbitration by a third review author. The Cohen’s kappa coefficient was used to assess the inter-rater agreements between authors for study selection and inclusion [23].

#### Appraisal of the methodological quality of included studies

The methodological quality of included studies was assessed using the Newcastle-Ottawa Scale version for cohort studies [24]. Considering that there is no validation study that provides a cut-off score for rating low-quality studies, we arbitrarily established that a study rated 0–3, 4–6, or 7–9 points would be considered at high, moderate, or low risk of bias, respectively. Two authors independently assessed the study quality, with disagreements resolved by consensus.

#### Data extraction and management

We used a preconceived and standardized extraction form to collect our data from each study, conducted independently by two authors. Disagreements between these authors were reconciled through discussion and consensus, or arbitration by another author. The Cohen’s kappa coefficient was used to assess the inter-rater agreements between authors for data extraction [23]. Data were abstracted from each study for the following elements: first author, year of publication, study objective(s) and design, period of data collection, country of study, setting, number of sites, sampling method, definitions used for current/former/ex/never-smoking, method of smoking assessment, period of smoking patterns measurement, mean/median duration of HIV, type of HIV, proportion on ART, mean/median duration of ART, mean/median age, age range, proportion of males, mean/median CD4 counts at study initiation/completion, mean/median viral load at study initiation/completion, mean/median duration of follow-up, total sample size/person-time of follow-up, number of PLWH with changes in smoking patterns, and study conclusion(s).

#### Data synthesis including assessment of heterogeneity

Meta-analysis was performed using ‘*meta*’ packages in R version 3.6.1 (*R Core Team, R foundation for Statistical Computing, Vienna, Austria*). We pooled the study-specific estimates using a random-effects meta-analysis model to obtain an overall summary estimate of the prevalence and mean across studies, after Freeman-Tukey double arc-sine transformation [25]. Estimates were reported with their 95% confidence interval (95% *CI*) and 95% prediction interval. Heterogeneity was assessed using the *χ*^2^ test on Cochrane’s Q statistic [26], and quantified by calculating the *I*^2^ [27]. Values of 25, 50 and 75% for *I*^2^ represented low, medium and high heterogeneity, respectively. We assessed the presence of publication bias using the formal Egger’s test [28]. A *P*-value < 0.10 was considered indicative of statistically significant publication bias.

## Results

### Study selection

Initially, a total of 7135 records were identified. After exclusion of duplicates and screening titles and abstracts, 7114 records were found irrelevant and then excluded. The inter-rater agreements between review authors for study inclusion and data abstraction remained high: κ = 0.94 and κ = 0.97, respectively; *P* < 0.001. Full-texts of the remaining 21 records were scrutinized for eligibility, among which 12 were excluded. In the end, nine studies were retained for the meta-analysis [19, 29–36] (Figure S1).

### Methodological quality and characteristics of included studies

Four studies had a low risk of bias, five studies had a moderate risk of bias, and no study had a high risk of bias. In total, 25 502 participants were included. All studies were prospective. Participants’ inclusion and follow-up ranged between 1984 and 2014. Studies were conducted in USA (*n* = 5), Canada (*n* = 2), Uganda (*n* = 1), and Switzerland (*n* = 1). Seven studies were multisite and two were single site. Six studies were hospital-based, two were population-based, and one did not specify the site. History of smoking was assessed by self-reporting in eight studies and by measuring nicotine in blood in one study. As reported in three studies [30, 31, 35], the mean duration between HIV diagnosis and inclusion in the original study varied from 10.5 to 15.2 years. The proportion of people on ART varied from 36 to 100% in six studies. The mean age of participants varied from 35 to 53 years. The proportion of male participants varied from 31 to 100%. The mean/median CD4 cells count varied from 133 to 496 cells/mm^3^ from five studies. The proportion of PLWH with detectable viral load at study initiation varied from 18 to 70% from three studies. The median duration of follow-up varied from 1.7 to 9.6 years from six studies. Counselling for tobacco smoking cessation was performed only in one study (Table S3).

### Smoking change patterns

**Fig. 1:**
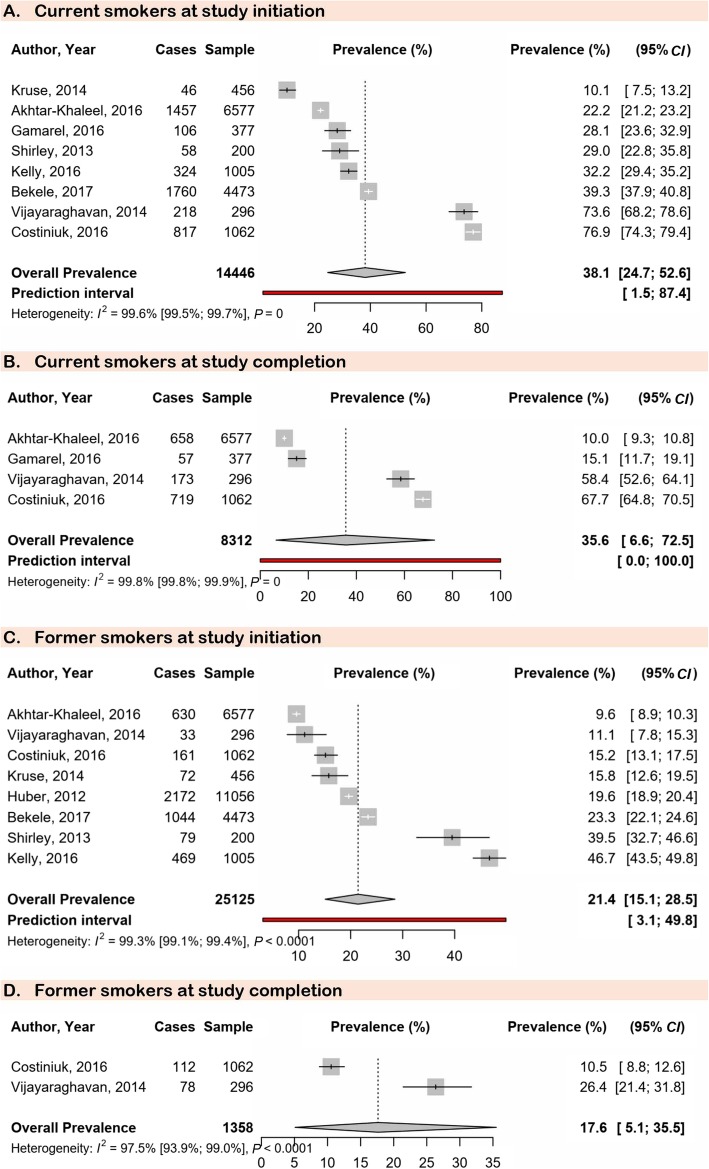
Meta-analysis prevalence of current and former smokers at study initiation and completion in the global population living with HIV. HIV: Human immunodeficiency virus

**Fig. 2:**
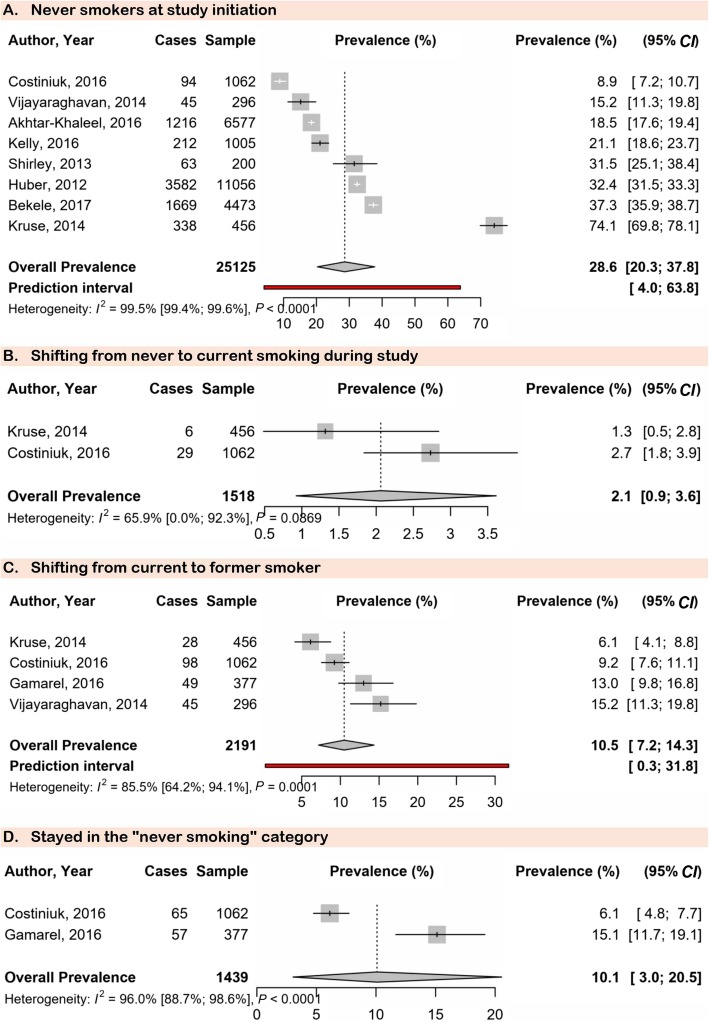
Meta-analysis prevalence of never and shifting smokers in the global population living with HIV. HIV: Human immunodeficiency virus

Meta-analysis results are presented in Table 1. The prevalence of current smokers at study initiation (38.1, 95% *CI*: 24.7–52.6) was in the range of the prevalence at the end of the study (35.6, 95% *CI*: 6.6–72.5) (Fig. 1). In the same way, the prevalence of former smokers at study initiation (21.4, 95% *CI*: 15.1–28.5) was in the range of the prevalence at the end of the study (17.6, 95% *CI*: 5.1–35.5) (Fig. 1). The prevalence of never smokers decreased from study initiation (28.6, 95% *CI*: 20.3–37.8) to study completion (6.1, 95% *CI*: 4.8–7.6), although only one study was included for study completion (Fig. 2). There was a low prevalence of people starting smoking during the study: shifting from never to current smoking (2.1, 95% *CI*: 0.9–3.6) and from former to current smoking (1.5, 95% *CI*: 0.6–2.9) (Fig. 2). The prevalence of shifting from current to former smoker was 10.5% (95% *CI*: 7.2–14.4) (Fig. 2). The prevalence for staying in the never smoking category was 10.1% (95% *CI*: 3.0–20.5) (Fig. 2). Substantial heterogeneity was found, however, there was no publication bias for all analyses (Table 1).

**Table 1:** Patterns of tobacco smoking change in people living with HIV

	Prevalence (95% *CI*)	95% Prediction interval	*N* studies	*N* participants	Heterogeneity			*P* Egger test
					H (954% *CI*)	*I*^2^ (95% *CI*)	*P* value	
Current smokers at study initiation	38.1 (24.7–52.6)	1.5–87.4	8	14 446	15.7 (14.2–17.3)	99.6 (99.5–99.7)	< 0.0001	0.458
Current smokers at study completion	35.6 (6.6–72.5)	0.0–100	4	8312	24.2 (21.7–27.0)	99.8 (99.8–99.9)	< 0.0001	0.321
Former smokers at study initiation	21.4 (15.1–28.5)	3.1–49.8	8	25 125	11.7 (10.4–13.2)	99.3 (99.1–99.4)	< 0.0001	0.528
Former smokers at study completion	17.6 (5.1–35.5)	NA	2	1358	6.4	97.5 (93.9–99.0)	< 0.0001	NA
Never smokers at study initiation	28.6 (20.3–37.8)	4.0–63.8	8	25 125	14.0 (12.6–15.5)	99.5 (99.4–99.6)	< 0.0001	0.930
Never smokers at study completion	6.1 (4.8–7.6)	NA	1	1062	NA	NA	NA	NA
Shifting from never to current smoking during study	2.1 (0.9–3.6)	NA	2	1518	1.7	65.9 (0.0–92.3)	0.087	NA
Shifting from former to current smoking during study	1.5 (0.6–2.9)	NA	1	456	NA	NA	NA	NA
Stopped smoking during the study (shifting from current to former smoker)	10.5 (7.2–14.4)	0.3–31.8	4	2191	2.6 (1.8–4.1)	85.5 (64.2–94.1)	0.0001	0.520
Stayed in the “never smoking” category	10.1 (3.0–20.5)	NA	2	1439	5.0	96.0 (88.7–98.6)	< 0.0001	NA
Tried to quit smoking but failed	7.1 (4.4–10.3)	NA	1	296	NA	NA	NA	NA
Willed to quit smoking but did not initiate any quitting attempt	0.4 (0.0–1.3)	NA	1	456	NA	NA	NA	NA

*HIV* Human immunodeficiency virus; *NA* not applicable; *CI* Confidence interval

## Discussion

This systematic review and meta-analysis aimed to investigate changes in smoking patterns among people living with HIV, globally. Although a high heterogeneity between studies was observed, the proportion of current and former smokers decreased slightly over time, around 2.5 and 3.8%, respectively. However, the proportion of never smokers decreased sharply by 22.5%, and there were 2.1 and 1.5% PLWH who shifted from never and former smoking to current smoking, respectively. On the other hand, 10.5% PLWH shifted from current to former smoking, 7.1% tried to quit tobacco consumption but failed, 10.1% stayed in the “never smoking” category over time and 0.4% willed to quit smoking but did not initiate any quitting attempt.

Clearly, the proportion of current and former smokers did not decrease significantly over time, despite the knowing harmful effects of tobacco smoking on PLWH’s health [3, 4, 7–11]. What’s worse, the proportion of never smokers decreased sharply over time, indicating perhaps that in the course of HIV-infection, a huge number of people started smoking. Actually, it has been argued that tobacco is being used among PLWH to cope with HIV-related symptoms such as neuropathic pains, anxiety, stress, and depression, all of which have been shown highly prevalent in this population [37, 38]. In addition, Mdege et al. bolstered that PLWH tend to express an inaccurate perception of their life expectancy which consequently affects their perceived susceptibility to the risk of tobacco use [18]. These facts brought the previous authors to suggest that dissemination of information on harmful effects of tobacco consumption might not be enough to reduce its use among PLWH [18].

Although it was reported from one study, the present review showed that no more than 0.4% PLWH willed to quit tobacco smoking but did not initiate any attempt. In accordance with the trans-theoretical model of health behaviour change used to describe the psychological mind-set of smokers [39], it can be inferred that most patients were still in the precontemplation phase while a minority of them might have reached the contemplation, preparation, action or even maintenance phase. Indeed, only 7.1% of PLWH tried to quit tobacco consumption but failed, corroborating other estimates driven from general smokers [40]. Kwong and Bouchard-Miller showed that quitting tobacco consumption is hard to reach and maintain because of both the addictive potential of nicotine and the withdrawal symptoms that accompany cessation [41]. Although there was no information about the reasons underlying these failures, one can easily suggest that these patients received no support and unsuccessfully tried to quit tobacco use by their own means [42, 43]. Accordingly, it has been demonstrated that smokers who had received adequate assistance and support towards smoking cessation had nearly doubled their success rate at 12 months in comparison to those who received no assistance [44].

This puts in light the paramount role that health care providers should play in helping PLWH to quit tobacco use, which commands that these care givers receive appropriate knowledge and competencies to deliver the right support. A recent review showed for instance that the success of a smoking-cessation intervention is also tributary of care providers’ knowledge and confidence to deliver the intervention [16]. Many types of healthcare providers are able to provide support towards smoking cessation including nurses, physicians, community and social workers, and health educators, all of whom are part of the HIV care givers’ team. It was shown that using multiple providers in a team approach (such as a physician and a nurse) could further enhance quit rates [41]. Therefore, tobacco use services ought to be integrated within HIV programs and be given full priority [18]. However, and considering the overwhelming task of managing HIV infection and its complications, smoking cessation may represent less of a priority from both providers’ and patients’ perspectives. Additionally, the heavy workload and shortages of health personnel which are particularly characteristic of low-income countries make time to be provided for extensive counselling very limited [45, 46]. These challenges need to be taken into consideration for the success of smoking cessation interventions in PLWH, especially in developing countries and sub-Sahara African countries. Indeed, 70% of PLWH reside in sub-Sahara Africa [6], a region with weak healthcare systems. Integration of tobacco use services in HIV programs and strengthening skills of HIV healthcare workers on strategies to help quitting tobacco consumption should be prioritized in this region as well as resources to be allocated for the task.

On the other hand and concerning what specific interventions should be implemented for PLWH who smoke, a review of tested interventions came to the conclusion that interventions may be more effective when its components are tailored to the unique needs of the target population [14]. For instance, depression and co-dependency on other substances such as alcohol and illicit drugs should be adequately addressed as part of a smoking cessation program for PLWH [16, 41]. In accordance with international guidelines, all trials implemented multifaceted interventions and utilized a combination of motivational interviewing/counselling techniques and pharmacotherapy (either nicotine-replacement therapy, nicotine agonists, or antidepressant therapy, unless pharmacotherapy is contraindicated) with varying degrees of intensity [16]. Other non-pharmacologic methods tested included telephone counselling, online quit programs, and cell phone counselling programs [16, 41]. Notwithstanding, it was emphasized the need for interventions that utilize multiple strategies and deliver highly intense interventions at multiple sessions [16, 41]. Moreover, effective interventions have to follow the “5 A’s” of smoking-cessation counselling, notably: ask about tobacco use, advise to quit, assess willingness to make a quit attempt, assist in quitting attempt, and arrange follow-up [16, 41].

Along with these specific interventions and in compliance with tobacco control policies derived from the WHO Framework Convention on Tobacco Control [47], some population-based interventions have yielded satisfactory outcomes towards reducing cigarette smoking and smoking-related disease and death, especially among subpopulations with the highest smoking prevalence. These strategies include tobacco price increases, comprehensive smoke-free laws, anti-tobacco mass media campaigns, and barrier-free access to tobacco cessation counselling and medications [48]. Definitely, there is crucial need for further research to better explore effective and cost-effective tobacco cessation interventions for PLWH that would be appropriate and scalable worldwide, especially in resource-constrained countries [18].

The present study should be interpreted in the context of some limitations. First, a substantial heterogeneity between studies included in the meta-analysis was observed, which is quite common to most systematic review of this kind and somewhat inevitable as well [49]. However, due to low number of studies, it was not possible to perform subgroup and meta-regression analyses to investigate sources of heterogeneity that may have influenced smoking pattern changes. These sources of heterogeneity might have included geographic, legal, cultural, clinical, socioeconomic, and demographic characteristics in different studies. Second, studies were disproportionally represented across various regions of the world, were not all population-based and nationally representative of PLWH; these facts may impede the translatability of these results to the entire globe. In addition, most studies were from high-income countries and only one study was from Africa, the epicentre of HIV infection with more than 70% of PLWH. It is therefore important to take this into account since findings of this study could more reflect the face of the public health concern in high income countries. This calls for more research in the context where it is most needed. Third, history of smoking was self-reported in these studies and some parameters were present in only one study, which may have introduced underestimation or overestimation of smoking status and changes. Nevertheless, and to the very best of the authors’ knowledge, this is the first systematic review and meta-analysis which has investigated changes in smoking behaviours among PLWH. A rigorous methodology was developed and robust statistical procedures were applied to examine the review’s research questions.

## Conclusions

This systematic review and meta-analysis revealed no positive changes in smoking patterns among people living with HIV, globally. These results put in light the crucial need to settle the fight against tobacco consumption as a priority in HIV care delivery in order to prevent the excess morbidity and mortality secondary to tobacco-related diseases in PLWH. Accordingly, cost-effective and appropriate interventions should be identified, that should be tailored to individual needs. Tobacco cessation services should be integrated in HIV programs and smoke-free policies should be implemented in HIV-treatment facilities. Having a pivotal role to play, HIV healthcare providers’ awareness and skills should be reinforced and upgraded to provide adequate cessation advice to patients and awareness of the harmful effects of smoking consumption and subsequent benefits of quitting particularly for PLWH should be increased. Beyond and considering the low number of studies included in this review, there is need to carry-out further well-designed researches to better investigate changes in smoking patterns in the course of HIV-infection.

## Supplementary information

**Additional file 1: Table S1.** MOOSE checklist **Table S2.** Search strategy in PubMed **Table S3.** Characteristics of included studies **Figure S1.** Process of identification and selection of studies for inclusion in the review (PRISMA flow diagram)

## Data Availability

Not applicable.

## Abbreviations

ART: Antiretroviral therapy
HIV: Human immunodeficiency virus
PLWH: People living with HIV
WHO: World Health Organisation
