# Supplementary material for: Changes in smoking patterns after HIV diagnosis or antiretroviral treatment initiation: a global systematic review and meta-analysis
Source: Infect Dis Poverty. 2020 Apr 16;9:35. doi: 10.1186/s40249-020-00644-z (PMC7160973; doi:10.1186/s40249-020-00644-z)
Supplement: Supplementary file 1 — Additional file 1: Table S1. MOOSE checklist Table S2. Search strategy in PubMed Table S3. Characteristics of included studies Figure S1. Process of identification and selection of studies for inclusion in the review (PRISMA flow diagram) [file 40249_2020_644_MOESM1_ESM.pdf]

Tobacco smoking changes in people living with HIV: a global systematic review and meta-analysis

Jobert Richie **Nansseu**, Dalhia Noelle **Tounouga**, Jean Jacques **Noubiap**, Jean Joel **Bigna**

APPENDIX

Table S 1. MOOSE checklist 2
Table S 2. Search strategy in PubMed 4
Table S 3. Characteristics of included studies... 5

Figure S 1. Process of identification and selection of studies for inclusion in the review (PRISMA flow diagram) 6

Table S 1. MOOSE checklist

Item No	Recommendation	Reported on Page No
Reporting of background should include		
1	Problem definition	4
2	Hypothesis statement	5
3	Description of study outcome(s)	4-5
4	Type of exposure or intervention used	NA
5	Type of study designs used	5
6	Study population	4-5
Reporting of search strategy should include		
7	Qualifications of searchers (eg, librarians and investigators)	6
8	Search strategy, including time period included in the synthesis and key words	6
9	Effort to include all available studies, including contact with authors	6
10	Databases and registries searched	6
11	Search software used, name and version, including special features used (eg, explosion)	6
12	Use of hand searching (eg, reference lists of obtained articles)	6
13	List of citations located and those excluded, including justification	6
14	Method of addressing articles published in languages other than English	6
15	Method of handling abstracts and unpublished studies	6
16	Description of any contact with authors	6
Reporting of methods should include		
17	Description of relevance or appropriateness of studies assembled for assessing the hypothesis to be tested	5
18	Rationale for the selection and coding of data (eg, sound clinical principles or convenience)	7-8
19	Documentation of how data were classified and coded (eg, multiple raters, blinding and interrater reliability)	7-8
20	Assessment of confounding (eg, comparability of cases and controls in studies where appropriate)	7-8
21	Assessment of study quality, including blinding of quality assessors, stratification or regression on possible predictors of study results	7
22	Assessment of heterogeneity	8
23	Description of statistical methods (eg, complete description of fixed or random effects models, justification of whether the chosen models account for predictors of study results, dose-response models, or cumulative meta-analysis) in sufficient detail to be replicated	8-9
24	Provision of appropriate tables and graphics	8-9
Reporting of results should include		
25	Graphic summarizing individual study estimates and overall estimate	9-10
26	Table giving descriptive information for each study included	9-10

27	Results of sensitivity testing (eg, subgroup analysis)	9-10
28	Indication of statistical uncertainty of findings	9-10

Reporting of discussion should include		
29	Quantitative assessment of bias (eg, publication bias)	14
30	Justification for exclusion (eg, exclusion of non-English language citations)	NA
31	Assessment of quality of included studies	14
Reporting of conclusions should include		
32	Consideration of alternative explanations for observed results	11-13
33	Generalization of the conclusions (ie, appropriate for the data presented and within the domain of the literature review)	11-13
34	Guidelines for future research	11-12
35	Disclosure of funding source	15

From: Stroup DF, Berlin JA, Morton SC, et al, for the Meta-analysis Of Observational Studies in Epidemiology (MOOSE) Group. Meta-analysis of Observational Studies in Epidemiology. A Proposal for Reporting. *JAMA*. 2000;283(15):2008-2012. doi: 10.1001/jama.283.15.2008.

Table S 2. Search strategy in PubMed

Search	Search terms
#1.	HIV or AIDS or “human immunodeficiency virus” or “acquired human immunodeficiency virus”
#2.	Smoking OR tobacco OR snuff OR cigarette OR Cigar OR pipe OR chewing OR Smoking OR nicotine OR tabacum OR nicotiana OR waterpipe OR e-cig OR e-cigarette
#3.	#1 AND #2
	Limits 2000/01/01 to 15/04/2018

Table S 3. Characteristics of included studies

Author	Year	Study period	Study design	Country	Number of sites	Site	Sampling	Timing of data collection	How was smoking history assessed?	Mean duration of HIV	%on antiretroviral	Mean age (years)	%Males	Mean/Median CD4 count (/ml) at study initiation	%Detectable viral load	Median duration of follow-up	Sample	Counseling for smoking cessation	Score, risk of bias
**Akhtar-Khaleel**	2015	1984-2012	Cohort study	USA	Multiple	Hospital-based	Consecutive	Prospective	Self-reported	NR	35.7	34.0	100	NR	70	9.6 years	6577	No	7
**Bekele**	2017	2008-2014	Cohort study	Canada	Multiple	Hospital-based	Random	Prospective	Self-reported	NR	85.6	45.3	81.7	NR	NR	3.5 years	4473	No	8
**Costiniuk**	2016	2003-2014	Cohort study	Canada	Multiple	Hospital-based	Consecutive	Prospective	Self-reported	10.5 years	NR	44	71	478	43	1.7 years	1062	No	6
**Gamarel**	2016	2009-2014	Cohort study	USA	Single	Population-based	Consecutive	Prospective	Self-reported	13.45 years	100	46.15	100	NR	NR	2 years	377	No	5
**Huber**	2012	2000-2010	Cohort study	Switzerland	Multiple	NR	NR	Prospective	Self-reported	NR	89	38	70	496	NR	NR	11056	Yes	6
**Kelly**	2016	1984-2003	Cohort study	USA	Multiple	Hospital-based	Random	Prospective	Self-reported	NR	NR	53.3	100	248	18	NR	1005	No	6
**Kruse**	2014	2005-2011	Cohort study	Uganda	Single	Hospital-based	Consecutive	Prospective	By measuring nicotine in blood	NR	100	35	31.1	133	NR	3.7 years	456	No	8
**Shirley**	2013	2012-2013	Cohort study	USA	Multiple	Hospital-based	Consecutive	Prospective	Self-reported	15.2 years	97	49	84	277	NR	NR	200	No	4
**Vijayaraghavan**	2013	2007-2008	Cohort study	USA	Multiple	Population-based	Systematic	Prospective	Self-reported	NR	NR	49.4	71.9	NR	NR	2 years	296	No	7

NR: Not reported

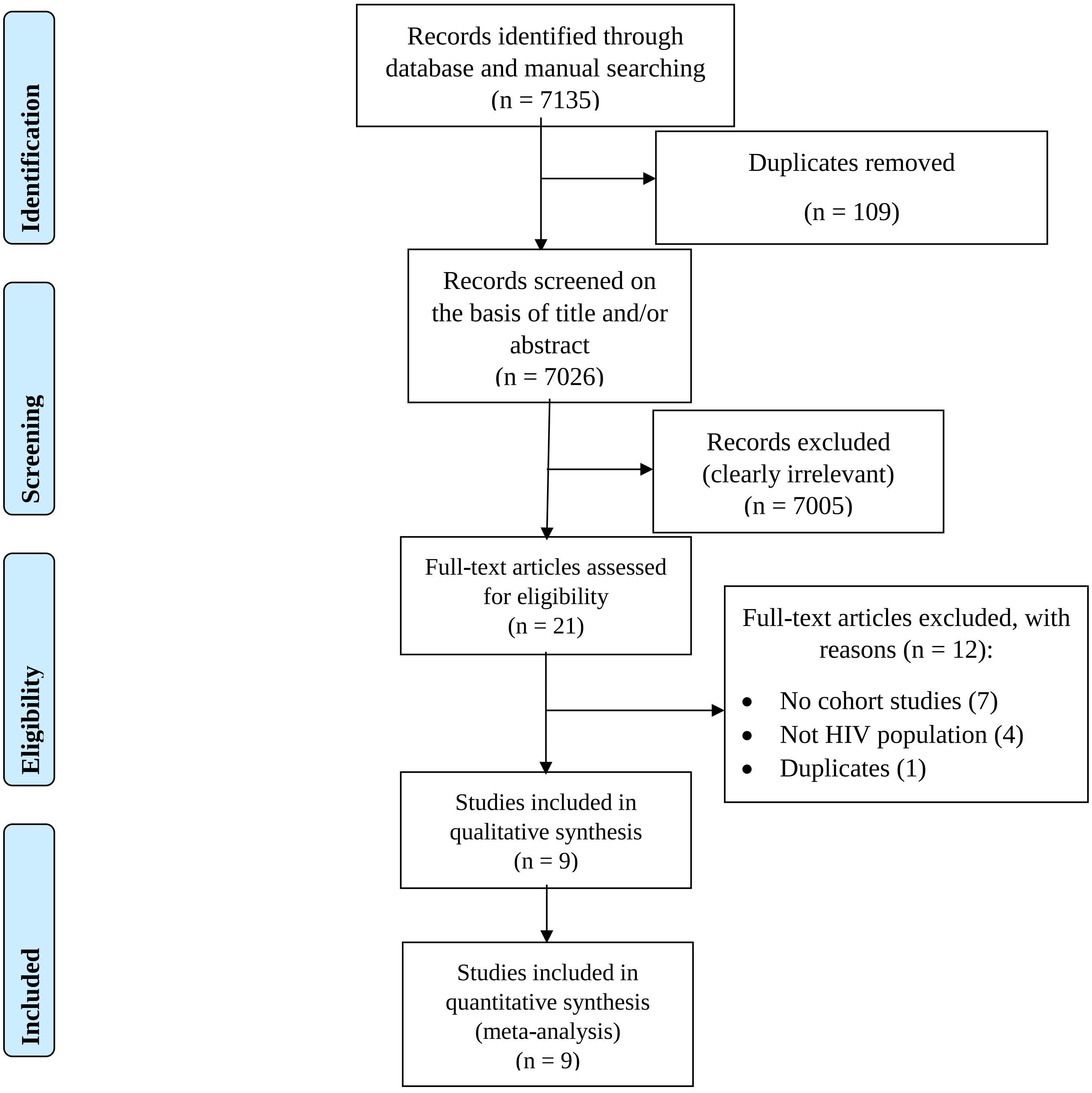

Figure S 1. Process of identification and selection of studies for inclusion in the review (PRISMA flow diagram)
